# Introducing an assessment tool based on a full set of end-of-training EPAs to capture the workplace performance of final-year medical students

**DOI:** 10.1186/s12909-019-1600-4

**Published:** 2019-06-13

**Authors:** Harm Peters, Ylva Holzhausen, Asja Maaz, Erik Driessen, Anja Czeskleba

**Affiliations:** 1 Dieter Scheffner Center for Medical Education and Educational Research, Dean’s Office of Student Affairs, Charité – Universitätsmedizin Berlin, Free University of Berlin and Humboldt University of Berlin, Berlin, Germany; 2 Department of Educational Development and Research, Faculty of Health Medicine and Life Sciences, Maastricht University, Maastricht, The Netherlands

**Keywords:** Final-year clerkship, Workplace performance, Assessment, EPAs

## Abstract

**Background:**

While literature on the theoretical value of entrustable professional activities (EPAs) for assessment is rapidly expanding, little experience exists on its application. The aims of this study are to develop and explore the utility of an EPA-based assessment tool for capturing the workplace performance of final-year medical students based on a full set of end-of-training EPAs.

**Methods:**

The tool was developed in a systematic iterative process. Twelve 12 end-of-undergraduate medical training EPAs were nested into 72 smaller EPAs and cross-mapped onto a 6-point supervision level scale, both adjusted to the context of final-year clerkships. One version was created for students’ self-assessment of their ability to carry out tasks and their history of carrying out tasks, and another version was created for supervisors’ assessment of students’ ability to carry out tasks. The tool was administered to final-year clerkship students and their clinical supervisors to explore its utility as an assessment approach. The results were analysed using descriptive and interferential statistics.

**Results:**

We enrolled a total of 60 final-year medical students. For 33 students, ratings were provided from one supervisor and for 27 students from two supervisors. With regard to the reliability and validity of the tool, students’ and supervisors’ ratings showed an overall good internal consistency as well as variability between and within the EPAs. Over the full EPA range, students rated their ability to perform a task slightly higher than their task performance history and slightly lower than the supervisors’ ratings. Students’ self-ratings of their ability to perform a task correlated with their history in performing the task. Supervisors’ ratings correlated among supervisors and not with students’ ratings. Concerning educational outcomes, supervisors’ average rating of students’ ability to perform the EPAs without direct supervision was 64%, and key findings being double-checked.

**Conclusions:**

This study introduces a tool that is adjusted to the final-year clerkship context and can assess the workplace performance of trainees based on a full set of end-of-training EPAs. Its utility characteristics suggest that the tool may be employed as a formative and outcome-aligned approach to the assessment of final-year students before entering into residency.

**Electronic supplementary material:**

The online version of this article (10.1186/s12909-019-1600-4) contains supplementary material, which is available to authorized users.

## Background

Entrustable professional activities (EPAs) have emerged as a new conceptual approach to the assessment of workplace performance in medical education [1]. While several reports outline in detail the breadth and depth of end-of-undergraduate medical training EPAs [2–6], little experience exists so far in regard to the assessment of EPAs in undergraduate medical education. This concerns both formative assessment to provide feedback for learners and to steer their learning as well as summative, high-stakes entrustment decisions about the learner [7]. The purpose of the present study is to develop and explore the characteristics of a tool for capturing the workplace performance of final-year clerkship students in relation to a full set of end-of-undergraduate-medical-training EPAs.

The EPA concept, introduced by ten Cate in 2005, re-conceptualises performance in the workplace in a unifying manner across the continuum of medical training [1, 8]. It builds upon three main constructs: 1) “professional activities”, i.e., authentic units of work to be carried out by a profession, 2) “levels of supervision”, i.e., the level of support that a trainee needs to carry out a task in a safe and effective manner, and 3) “entrustment decisions”, i.e., the assignment of supervision levels to trainees to carry out a particular professional activity. The last construct can serve as a declaration of trainees’ workplace proficiency and thereby connects the two former constructs. EPAs are supposed to be gradually allocated to trainees once they have reached the sufficient competence required to carry out the tasks. The EPA concept aims at providing a learning trajectory towards the independent practice of physicians, i.e., developing trainees’ proficiency to carry out all tasks characteristic of the discipline in an unsupervised form [1].

Using EPAs as a framework for assessment has its roots in what is referred to as the dilemma of workplace-based assessment in medical education [1, 9]. One side of this dilemma is that the reliability of assessment instruments is low when learners are assessed in real-world settings [9]. This is because workplace-based assessment is difficult to standardise by checklist approaches because cases are unpredictable and cannot be scheduled or repeated [10]. Physicians (the assessors) feel uncertain regarding the standards, methods and goals of such assessments, especially in handling abstract scales with ratings from “unsatisfactory” to “superior”. In addition, the results of these assessments are often overly positive about the trainees and show few assessor differences on the scales used [9]. The other side of this dilemma is that when the quality of assessment is improved by, for instance, placing the trainee and the assessor in an environment standardised for difficulty and content, the trainee’s competence in such a setting does not predict real workplace performance well [9]. The EPA concept offers a new approach to addressing the dilemma of workplace-based assessment. Its three main constructs (professional activities, supervision levels and entrustment decision) align better with the thinking and expertise of the clinical supervisors who actually carry out the workplace assessment [1, 11].

The assessment of workplace performance with EPAs is currently evolving and will likely encompass a broad spectrum of various methods and approaches. EPAs for entry into residency and entrustment rating scales have recently been shown to be of added educational value in evaluation as well as in assessment approaches based on the self-rating of postgraduate graduate residents or the ratings of supervisors and programme directors [12–15]. To extend these studies to the undergraduate setting, it would be necessary to break down the EPAs for entry into residency into smaller tasks and to employ an entrustment rating scale, both of which would then need to be aligned with the context of undergraduate medical education.

At our institution, the Charité – Universitätsmedizin Berlin (Charité) – a group of experienced faculty supervisors recently defined a set of EPAs for entry into residency [6, 16, 17]. These EPAs are currently implemented as end-of-training EPAs in the undergraduate medical programme. As part of this process, our institution sought to use these EPAs to develop an assessment tool that would allow an EPA-based evaluation of the workplace performance of medical students in their final-year clerkship. To date, there is to our knowledge no assessment instrument available that provides an overview of the workplace performance of medical students in their final-year clerkship in relation to a full set of end-of-training EPAs. Information derived from such an assessment instrument could be employed to 1) steer the individual learning of students and the teaching by their clinical educators in a meaningful manner, 2) prepare graduates better for their first days in residency and with responsibility in patient care, and 3) inform curriculum development based on the outcomes achieved and/or not achieved with the programme.

The aims of this article are to report on the development of an assessment tool based on a full set of end-of-undergraduate medical training EPAs and to explore its utility for assessment in a cohort of medical students in their final-year clerkship and their supervisors. For the utility exploration, we will refer to a framework provided by van der Vleuten [18]. We will focus on measures of reliability and validity and report aspects related to educational impact and feasibility.

## Methods

### Setting

The study was carried out at the Charité, Germany, and its affiliated teaching hospitals in 2016. The study protocol received approval by the local data protection office and the Ethical Board (No. EA4/096/16). Participation required informed consent.

The Charité undergraduate medical programmes encompass six years [17]. The final year consists of three clerkship rotations with 3.5-month placements. The goal of the final-year clerkship is that medical students actively participate in the clinical workplace under the instruction, supervision and responsibility of a physician [19]. The final-year clerkship placements are preceded by five years of undergraduate training in basic and clinical sciences. Two versions of the undergraduate curriculum are currently in place covering similar contents, one with a horizontal alignment and one with horizontal and vertical integration. In both programmes, patient-based learning involves comparable amounts of basic skills training and bedside teaching (the total here is approximately 500 h). Early clerkship placements cover 4 months. Before the students can enter the final-year clerkship, they must pass a summative, nationwide written state examination with 320 multiple choice questions to demonstrate sufficient knowledge in clinical medicine. During the final-year clerkship, there are currently no mandatory formative or summative workplace assessments in place, as these are not required by the national regulations for this period of training [19]. During their final-year clerkship placements, the students participate to various extents in the day-to-day medical care and management of real patients. To what degree the medical students can actually participate is decided by their clinical supervisors. According to the local legal framework, professional tasks of a physician can be delegated to medical students, but students’ findings and decisions need formal verification by the supervising physician.

After the final-year clerkship, the students must pass a moderately structured oral-practical state examination to formally graduate as physicians [19]. This summative examination involves 4 medical students and 4 experienced physicians, one from internal medicine, one from surgery and two from elective disciplines. The examination lasts at least 8 h (2 h for each student) and involves assessment of history and physical examination skills on real patients, case-based discussions and answering questions related to patient management in the four disciplines represented by the four physician assessors.

### Tool development

The tool to capture workplace performance was developed in a systematic, iterative process. In the first step, we formed a working group comprising members with expertise in clinical education, curriculum development and educational research to develop the assessment instrument. Our guiding principles were as follows: 1) The assessment tool should consist of a two-dimensional matrix, where the rows specify professional activities and the columns indicate the level of supervision. 2) The tool should have a one-page paper-and-pencil version and a digital file version. 3) One version will serve for self-evaluation by the students, and one version will serve for assessment by corresponding clinical supervisors. 4) The assessment tool should capture the students’ and supervisors’ ratings on the full spectrum of end-of-training EPAs. 5) The instrument should entail a break-down of the 12 Charité end-of-training EPAs into smaller nested professional activities according to the developmental stage of final clerkship students. 6) The size of the nested tasks (units of work) should secure formal confirmation of medical findings and decisions by a supervising physician before the clerkship student can go on to the next task. 7) The professional tasks should be described in a manner that allows sufficient understanding of the activity and the patient-related context. 8) The activity descriptions should use clear language about tasks and contexts and should avoid educational jargon. 9) The supervision levels should be suitable for undergraduate medical education. 10) Both the list of tasks and the levels of supervision should comply with the legal requirements for the participation of medical students in medical patient care, including double-checking of key findings by the supervising physician.

The two-dimensional, EPA-based assessment instrument was developed in an iterative process within the working group and with continuous referral to the literature. The starting point was a full set of 12 core EPAs for entering postgraduate training, as recently validated in a Delphi study at our institution [6]. The supervision levels were operationalised according to literature [7, 20], with two subscales for direct supervision and three subscales for indirect supervision. The wording was adjusted to the German language context. We purposely left out supervision level 1 (“allowed to observe, but not perform”). We instead incorporated the category “not able to perform”.

Table 1 shows the operationalisation of the supervision levels as a 6-point scale. For the self-rating of their ability to carry out a task, the students were prompted with “I can carry out the activity sufficiently certain ….” and then were asked to indicate the supervision level needed. For students’ self-rating of their history in performing a task, the students were prompted with “I carried out this task at least three times at the supervision level of (highest level of independence)” and then were asked to indicate the level of supervision on a 6-point scale. For supervisors’ rating of the students’ ability to carry out a task, the supervisors were prompted with “the student can carry out the activity sufficiently certain….” and then are requested to indicate the level of supervision on a 6-point scale.

**Table 1:** Operationalisation of the terms “I can”, “I did” and “the student can” (Table 1A) and of the supervision levels on a 6-point scale (Table 1B) as used in the study. The third column of Table 1B indicates how the supervision scale used in this study relates to the Chen-scale for undergraduate medical education (Chen et al., 2015)

Table 1A: Term	Operationalisation of term in this study	
“I can”	Student´s self-rating on the ability to carry out a task	
“I did”	Student´s self-rating on own history having carried out a task	
“The student can”	Supervision´s rating on the ability of a student to carry out a task	
Table 1B: Scale	Operationalisation of supervision levels	Levels according to Chen-scale [20]
1	Cannot carry out the activity.	0
2	Act in co-activity with the supervisor.	2a
3	Act on one's own while the supervisor is present and steps in if needed.	2b
4	Act on one's own with supervision available within minutes (supervisor on the ward) and *all* findings are double-checked.	3a
5	Act on one's own with supervision available within minutes (supervisor on the ward) and *key* findings are double-checked.	3b
6	Act on one's own with supervision available distantly (e.g. by phone) and *key* findings are double-checked.	3c

The developed assessment tool entails a total of 72 nested professional activities combined in 12 end-of-training EPAs, all organised into 5 EPA domains. The 6-point supervision scale is not applicable to 4 of the 72 nested EPA because the activities are performed together with or in the presence of the supervisor (all in EPA domain 1). The EPA-based assessment instrument was pilot tested in a paper-and-pencil version with a total of 3 final-year clerkship students and 2 of their supervisors, who provided oral and written feedback. The iterations and pilot testing led to the refinement of the assessment form. This included improving the language and specifying the context. Complete versions of the EPA-based assessment instruments for students and supervisors are enclosed in the Additional files 1 and 2.

### Tool application

The instrument was administered first to final-year clerkship students and subsequently to their supervisors. Ratings were not shared between the students and supervisors. Students could voluntarily participate in the study at the end of their second rotation of the final-year clerkship and received financial compensation. For resource reasons, a maximum of 60 students could be included. We purposely chose the end of the second rotation to allow for a long period of contact between students and supervisors. We omitted the third rotation because we had expected an insufficient participation rate due to the final state examination, which is taken after this rotation.

In the first study phase, the study purpose was introduced to groups of clerkship students in a classroom setting as a general evaluation without any standard setting, i.e., without any task performances or supervision levels to be met. After receiving brief instruction, students individually completed a paper-and-pencil version of the instrument. They were individually asked to provide information on one or more clerkship supervisors to be approached for co-assessment.

In the next phase, the study’s purpose and procedure were introduced individually to supervisors by phone as a general evaluation of final clerkship students without any standard setting. They were informed that their assessment was only for study purposes, would not be given to the students and would not have any consequence for the formal assessment of the individual students. In the case of two supervisors for one student, they were each asked to fill out the assessment form independently. After a brief introduction, they received a digital, Excel®-based version of the instrument, which was to be filled out and sent back. The supervisors did not receive any compensation for their time invested. When instructing the students and supervisors, we did not explicitly refer to the EPA concept. In our language, we used “evaluation of clinical competence”, as expressed by a matrix listing the clerkship students’ tasks and the levels of supervision.

### Utility evaluation of the assessment tool

According to the stage of development, we decided on an explorative utility evaluation of the tool for assessment purposes. This utility evaluation is based on a framework introduced by van der Vleuten to measures of feasibility, acceptability, reliability, validity and educational value [18]. In our study, reliability and validity measures are based on descriptive and interferential statistics of ratings by students and supervisors on the EPAs. Educational value in this study refers to educational outcomes met by the programme, i.e., to the percentage of students who reach at least a certain level of supervision in the EPA performance. For programme directors and curriculum managers, this is relevant educational information, i.e., on the appropriateness of the spectrum of tasks and the level of supervision, which is an outcome goal for their undergraduate medical training programme. The intended outcome goal at the Charité is that students in the final-year clerkship reach at least supervision level 5 in the end-of-training EPAs, except for cases where direct supervision is legally required. For US EPAs, the general graduation expectation is that graduates are able to perform the tasks without direct supervision, and this corresponds to level 4 or higher in our scale [3]. As this utility evaluation is explorative in nature, we refer to both levels of educational outcomes met by students in our analysis of the study results. To obtain insights into the feasibility of this tool, we report estimates of the costs, logistics, equipment and resources that it requires. The acceptability of the tool cannot be derived in this study because the students were voluntarily selected and financially compensated.

### Statistical analysis

Descriptive and interferential statistical analyses were carried out using SPSS 23 (IBM, Ehningen, Germany) and JASP 0.8.6 (JASP Team, 2018). The results are expressed as the mean ± standard deviation. Significance levels are set at the *p* < 0.05 level.

Using descriptive statistics, we report the variability of the ratings within and between EPAs for the three ratings “I can”, “I did” and “the student can”. Descriptive statistics were also used to report the utility aspects of the feasibility and educational value of the assessment tool. For the latter, supervisor ratings were used to calculate the mean percentage of students who reached at least level 4 and the percentage of those who reached at least level 5 for each of the 12 EPAs based on averaging the results of the nested EPA subscales.

Measures of reliability and validity were explored using interferential statistics. The reliability of the subscales for the 12 EPAs was calculated using McDonald’s omega. To test for group differences between the three ratings, we employed a linear mixed model (LMM) approach for repeated measures. In cases where there were two supervisors’ ratings, the mean was calculated before further analysis was undertaken. The subject ‘student’ represented the upper level of hierarchy; the three corresponding EPA ratings were set as the lower level (fixed effect). The LMM approach was chosen because it allows the processing of data sets with randomly missing values, and it can also be extended to data that are not normally distributed [21]. We assumed that the covariance between random errors was not completely independent; therefore, we estimated the residual covariance structure as compound symmetry with heterogeneous variances. In the case of significant results in the LLM analysis, least significant difference was used as a post hoc test, and the effect size (Cohen’s d) was calculated. Furthermore, we performed correlation analyses between the three ratings using Pearson’s r. When correlating a student’s self-rating with a supervisor’s rating, the mean was used in the case of two supervisors. When correlating the ratings between supervisors, the individual supervisor ratings were correlated with each other.

## Results

### Tool application

Sixty students participated in the study; 34 were females, and 26 were males. This ratio is comparable to that of the whole student cohort at the Charité. The participants represented 16.4% of the 364 students invited. Assessments were requested from 94 supervisors, and 87 supervisors took part, including 36 females and 51 males. For 33 students, ratings were provided by one supervisor and for 27 students by two supervisors. Ninety-six percent of the clerkship placements were in hospitals (68% in non-university and 28% in university hospitals), and 4% were in primary care.

In the following, we report on the exploration of the utility characteristic of the assessment tool.

### Utility measures of reliability and validity

**Fig. 1:**
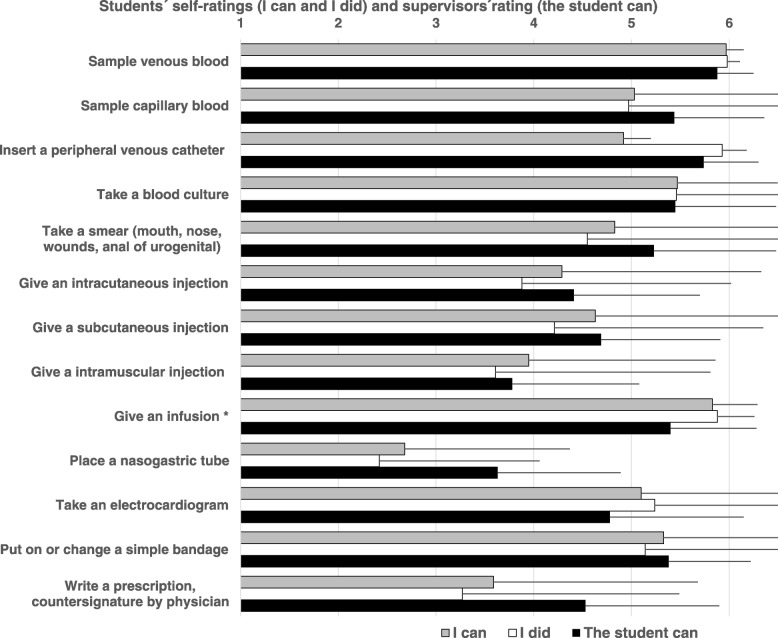
Clerkship students’ self-ratings on the ability to perform a task (I can) and the history in performing a task (I did) as well as the supervisors’ rating on the students´ ability to perform the task (the student can) in relation to a 6-point supervision level scale (1–6). Shown are the results of 13 task of EPA 2. Perform general procedures of a physician. The bars depict the mean plus standard deviation. Significant differences were found in one of the 13 medical procedures (give an infusion, Mixed model, F(2, 65.038) = 5.190, *p* = 0.008, I can versus I did: *p* < 0.003, d = − 0.075, I can versus the student can: *p* = 0.003, d = − 0,494)

Table 2 and Fig. 1 depict the results of the three ratings across the scales and range of the 12 EPAs. Students’ and supervisors’ ratings show an overall variability between and within the EPAs. Students rated their ability to carry out a task highest in EPAs 1.1 and 3.1 and lowest in EPAs 5.1 and 5.2. These ratings mirror their history in carrying out the tasks. Supervisors rated students’ ability to carry out a task highest in EPAs 2 and 4.1 and lowest in EPAs 3.1 and 5.1. Over the 12 EPAs, the students rated their ability to perform a task (4.69 ± 0.39) higher than they rated their history in performing a task (4.37 ± 0.64), with a mean difference of 0.32 (range 0.00 to 1.06). Supervisors rated students’ ability to perform a task slightly higher (4.82 ± 0.16), with a mean difference of 0.13 (range − 0.30 to 0.88) compared to the students’ self-ratings.

**Table 2:** Clerkship students´ self-ratings on the ability to perform a task (I can) and the history in performing a task (I did) as well as the supervisors’ rating on the students´ ability to perform the task (the student can) in relation to a 6-point supervision level scale (1–6) for 12 end-of-training EPAs. The numbers in parentheses indicate the number of subscales. Mean group ratings are compared by a linear mixed model followed by post hoc last significance differences (LSD) testing

		Students		Supervisors	Overall group comparison
		I can	I did	The student can	Liniar mix model
		Mean ± SD	Mean ± SD	Mean ± SD	
1.	Along the clinical encounter				
1.1.	Take a medical history, perform a physical examination and provide a structured summary of the results (6)Take a medical history, perform a physical examination and provide a structured summary of the results (6)	5.23 ± 0.54	5.23 ± 0.67	4.93 ± 0.67	F(2, 79.601) = 4.631, *p*=0.013
1.2.	Compile a diagnostic plan and initiate implementation (5)	4.97 ± 0.85	4.77 ± 0.92	4.79 ± 0.81	F(2, 76.903) = 1.699, *p*=0.190
1.3.	Interpret test results and initiate further steps (6)	4.76 ± 0.86	4.69 ± 1.04	4.70 ± 0.89	F(2, 77.258) = 0.345, *p*=0.710
1.4.	Compile a treatment plan and initiate implementation (5)	4.80 ± 0.93	4.65 ± 1.17	4.71 ± 0.91	F(2, 80.601) = 0.730, *p*=0.485
2.	Perform general procedures of a physician				
	Procedures (13)	4.82 ± 0.76	4.65 ± 0.86	4.96 ± 0.81	F(2, 77.255) = 2.662, *p*=0.076
3.	Communication with patients				
3.1.	Seek consent for medical procedures and diagnostics (2)	5.01 ± 0.97	4.80 ± 0.99	4.65 ± 1.09	F(2, 71.818) = 3.227, *p*=0.045
3.2.	Inform and advise a patient (6)	4.72 ± 1.04	4.19 ± 1.31	4.90 ± 0.96	F(2, 81.382) = 5.618, *p*=0.005
4.	Communication with colleagues				
4.1.	Present a patient history (2)	4.91 ± 1.36	4.56 ± 1.58	5.04 ± 1.06	F(2, 94.993) = 2.879, *p*=0.061
4.2.	Give or receive a patient handover (2)	4.48 ± 1.50	4.09 ± 1.82	4.70 ± 1.07	F(2, 98.756) = 3.729, *p*=0.027
4.3.	Compile and distribute a patient report (3)	4.66 ± 1.08	4.51 ± 1.33	4.83 ± 0.91	F(2, 85.883) = 1.422, *p*=0.247
5.	Further professional activities of a physician				
5.1.	Recognize an emergency situation and act upon it (13)	3.73 ± 1.19	2.67 ± 1.28	4.51 ± 1.21	F(2, 74.859) = 32.315, p<0.001
5.2.	Undertake an evidence-based patient case presentation and initiate patient-specific implementation (5)	4.18 ± 1.40	3.63 ± 1.63	5.06 ± 0.86	F(2, 91.474) = 25.137, p<0.001

Table 3 shows the results of the McDonald’s omega calculations for the internal consistency of the rating of the subscales of the 12 end-of-training EPAs. The internal reliability is good for 11 of the 12 EPAs. It is considered insufficient for EPA 3.1 (below 0.7) in relation to both the students’ and supervisors’ ratings. The results on this EPA are not withdrawn from further analyses because of the explorative intent of this study.

**Table 3:** Internal consistency of the clerkship students´ and supervisors´ ratings on 12 end-of-training EPAs. Scale reliabilities are calculated by McDonald’s omega. The numbers in parentheses indicate the number of subscales

		Students		Supervisors
		I can	I did	The student can
1.	Along the clinical encounter			
1.1.	Take a medical history, perform a physical examination and provide a structured summary of the results (6)Take a medical history, perform a physical examination and provide a structured summary of the results (6)	0.774	0.789	0.800
1.2.	Compile a diagnostic plan and initiate implementation (5)	0.802	0.745	0.825
1.3.	Interpret test results and initiate further steps (6)	0.780	0.846	0.841
1.4.	Compile a treatment plan and initiate implementation (5)	0.854	0.837	0.850
2.	Perform general procedures of a physician			
	Procedures (13)	0.725	0.728	0.912
3.	Communication with patients			
3.1.	Seek consent for medical procedures and diagnostics (2)	0.190	0.147	0.605
3.2.	Inform and advise a patient (6)	0.771	0.834	0.941
4.	Communication with colleagues			
4.1.	Present a patient history (2)	0.811	0.793	0.920
4.2.	Give or receive a patient handover (2)	0.780	0.848	0.930
4.3.	Compile and distribute a patient report (3)	0.598	0.741	0.892
5.	Further professional activities of a physician			
5.1.	Recognize an emergency situation and act upon it (13)	0.937	0.936	0.977
5.2.	Undertake an evidence-based patient case presentation and initiate patient-specific implementation (5)	0.865	0.854	0.905

When comparing the rating results with group comparisons, the statistical analyses indicated significant group differences for only 6 of the 12 EPAs (Table 3). In the post hoc analyses, students rated their ability to perform a task significantly higher (3 of the 12 EPAs) than their history in performing the task (3.2: *p* = 0.007; d = 0.514), 5.1: *p* < 0.001; d = 0.871 and 5.2: *p* = 0.019; d = 0.497). Significant differences between students’ and supervisors’ ratings in post hoc analyses were seen in 4 of the 12 EPAs. Students rated their ability to perform a task themselves higher in EPA 1.1 (*p* = 0.006; d = 0.377), EPA 2.9 (*p* = 0.003; d = − 0.494) and EPA 3.1 (*p* = 0.017; d = 0.274), while supervisors rated the students’ ability higher in EPA 5.1 (*p* = 0.001; d = − 0.401) and 5.2 (p < 0.001; d = − 0.532).

In further interferential analysis, students’ ratings of their ability to perform a task correlate closely with their history in performing the task. Significant correlations are seen in 11 EPAs and in 11 of the 13 general procedures of a physician (Table 4). The students’ self-rated ability to perform a task does not correlate with the supervisors’ ratings. The ratings of two supervisors on one student’s ability to perform a task correlate significantly in 8 of the 12 EPAs.

**Table 4:** Correlations between Clerkship students´ self-ratings on the ability to perform a task (I can) and the history in performing a task (I did) as well as the supervisors rating on the students´ ability to perform the task (the student can, individual [1 and 2] and mean of two supervisors [mean]) in relation to a 6-point supervision level scale. Shown are the results for 12 end-of-training EPAs, including the tasks of EPA 2. The numbers in parentheses indicate the number of subscales. Correlations are calculated by Pearson´ r

		I can	I can	Student can 1
		I did	Student can_mean	Student can 2
1.	Along the clinical encounter			
1.1.	Take a medical history, perform a physical examination and provide a structured summary of the results (6)Take a medical history, perform a physical examination and provide a structured summary of the results (6)	0.635, *p* <0.000	0.114, *p*=NS	0.339, *p*=NS
1.2.	Compile a diagnostic plan and initiate implementation (5)	0.563, *p* <0.000	-0.049, *p*=NS	0.344, *p*=NS
1.3.	Interpret test results and initiate further steps (6)	0.709, *p* <0.000	-0.053, *p*=NS	0.576, *p* <0.01
1.4.	Compile a treatment plan and initiate implementation (5)	0.641, *p* <0.000	0.025, *p*=NS	0.748, *p* <0.001
2.	Perform general procedures of a physician			
2.1.	Sample venous blood	-0.017, *p*=NS	0.0070, *p*=NS	0.744, *p* <0.000
2.2.	Sample capillary blood	0.950, *p* <0.000	0.167, *p*=NS	0.111, *p*=NS
2.3.	Insert a peripheral venous catheter	0.195, *p*=NS	-0.037, *p*=NS	0.763, *p* <0.000
2.4.	Take a blood culture	0.832, *p* <0.000	0.540, *p*=NS	0.863, *p* <0.000
2.5.	Take a smear (mouth, nose, wounds, anal of urogenital)	0.793, *p* <0.000	-0.139, *p*=NS	0.633, *p* <0.001
2.6.	Give an intracutaneous injection	0.878, *p* <0.000	-0.170, *p*=NS	0.491, *p* <0.01
2.7.	Give a subcutaneous injection	0.875, *p* <0.000	0.008, *p*=NS	0.118, *p*=NS
2.8.	Give a intramuscular injection	0.841, *p* <0.000	-0.161, *p*=NS	-0.111, *p*=NS
2.9.	Give an infusion	0.403, *p* <0.01	0.200, *p*=NS	0.523, *p* <0.01
2.10.	Place a nasogastric tube	0.808, *p* <0.000	0.194, *p*=NS	0.540, *p* <0.01
2.11.	Take an electrocardiogram	0.907, *p* <0.000	0.202, *p*=NS	0.608, *p* <0.01
2.12.	Put on or change a simple bandage	0.801, *p* <0.000	0.144, *p*=NS	0.360, *p*=NS
2.13.	Write a prescription, countersignature by physician	0.826, *p* <0.000	0.034, *p*=NS	0.178, *p*=NS
3.	Communication with patients			
3.1.	Seek consent for medical procedures and diagnostics (2)	0.820, *p* <0.000	0.025, *p*=NS	0.582, *p* <0.01
3.2.	Inform and advise a patient (6)	0.548, *p* <0.000	-0.036, *p*=NS	0.433, *p* <0.05
4.	Communication with colleagues			
4.1.	Present a patient history (2)	0.677, *p* <0.000	0.342, *p*=NS	0.395, *p* <0.05
4.2.	Give or receive a patient handover (2)	0.643, *p* <0.000	0.198, *p*=NS	0.090, *p*=NS
4.3.	Compile and distribute a patient report (3)	0.798, *p* <0.000	-0.214, *p*=NS	0.603, *p* <0.01
5.	Further professional activities of a physician			
5.1.	Recognize an emergency situation and act upon it (13)	0.478, *p* <0.000	-0.029, *p*=NS	0.712, *p* <0.000
5.2.	Undertake an evidence-based patient case presentation and initiate patient-specific implementation (5)	0.603, *p* <0.000	-0.006, *p*=NS	0.653, *p* <0.000

### Utility aspects related to educational value

Based on the supervisors’ ratings for the 12 EPAs, a mean of 85% of the students reached supervision level 4 or higher, and a mean of 64% reached supervision level 5 or higher (Table 5). In the tasks of EPA 2 (“general procedures of a physician”), these mean values were 81 and 66%, respectively.

**Table 5:** Mean percentage of students who reach supervision level 4 or higher (left) as well as level 5 and higher (right) in performing the tasks on the supervisor’s ratings for 12 end-of-training EPAs, including the task in EPA 2. The numbers in parentheses indicate the number of subscales

		Level 4	Level 5
		and higher	and higher
1.	Along the clinical encounter		
1.1.	Take a medical history, perform a physical examination and provide a structured summary of the results (6)Take a medical history, perform a physical examination and provide a structured summary of the results (6)	89%	68%
1.2.	Compile a diagnostic plan and initiate implementation (5)	85%	61%
1.3.	Interpret test results and initiate further steps (6)	84%	61%
1.4.	Compile a treatment plan and initiate implementation (5)	83%	57%
2.	Perform general procedures of a physician		
2.1.	Sample venous blood	100%	98%
2.2.	Sample capillary blood	94%	85%
2.3.	Insert a peripheral venous catheter	100%	92%
2.4.	Take a blood culture	89%	81%
2.5.	Take a smear (mouth, nose, wounds, anal of urogenital)	85%	79%
2.6.	Give an intracutaneous injection	67%	44%
2.7.	Give a subcutaneous injection	78%	51%
2.8.	Give a intramuscular injection	49%	24%
2.9.	Give an infusion	94%	83%
2.10.	Place a nasogastric tube	50%	21%
2.11.	Take an electrocardiogram	81%	64%
2.12.	Put on or change a simple bandage	94%	83%
2.13.	Write a prescription, countersignature by physician	75%	53%
3.	Communication with patients		
3.1.	Seek consent for medical procedures and diagnostics (2)	75%	63%
3.2.	Informe and advise a patient (6)	84%	66%
4.	Communication with colleagues		
4.1.	Present a patient history (2)	89%	74%
4.2.	Give or receive a patient handover (2)	82%	59%
4.3.	Compile and distribute a patient report (3)	92%	57%
5.	Further professional activities of a physician		
5.1.	Recognize an emergency situation and act upon it (13)	78%	60%
5.2.	Undertake an evidence-based patient case presentation and initiate patient-specific implementation (5)	91%	72%

### Utility aspect related to feasibility

The costs of test administration are low for the paper version and the electronic-file-based version of the assessment form. Test administration involves some staff resources for handing out and monitoring the completion and collection of the assessment form and subsequent analysis. Students and supervisors need only a brief introduction on how to use the instrument. Students need approximately 30 min and supervisors approximately 20 min to complete the assessment form.

## Discussion

Approaches to assessment based on EPAs represent a current and active field of research and development in medical education. This study introduces an assessment tool that can capture the workplace performance of medical students in the final-year clerkship in relation to a full set of EPAs serving as outcomes for undergraduate medical education. In the following, we discuss the development and the utility exploration of this assessment tool in light of the current literature.

The EPA-based assessment tool was developed in a systematic, iterative process with the intention to closely represent the real-life workplace participation of medical students in their final-year clerkship. For this process, it was important to adapt the granularity of professional activities and supervision levels to the conditions in the final-year clerkship of undergraduate medical education in our context. A number of guiding principles were established for this process. The 12 end-of-undergraduate-training EPAs, as defined at our institution, were broken down into 72 nested EPAs, i.e., all sub-tasks (units of work) that can separately be carried out, observed and assessed. We developed a 6-point supervision scale for undergraduate medical education closely referencing the published literature [7, 20, 22]. We believe that the report on our approach can help orient and guide other medical faculties that consider developing similar EPA-based assessment approaches for their students in the final-year clerkship. We also believe that the assessment tool introduced in this report allows a tangible overview of the workplace performance of final-year clerkship students based on a full range of end-of-undergraduate-training EPAs. This view is of particular importance because final-year students are in the transition from undergraduate to postgraduate medical training and are expected to be proficient in performing these EPAs at a higher degree of autonomy in the very near future.

When the EPA-based assessment tool was administered to a group of final-year clerkship students and their supervisors, we were able to explore several utility characteristics of the tool. The statistical analysis of the ratings by students and supervisors contributed to insights into the reliability and validity evaluation of the assessment tool. In the following paragraph, we elaborate on the distribution of the ratings, the consistency of the ratings on the subscales and the comparison and correlations between the three groups of ratings.

Considering the distribution of the ratings by students and supervisors, the tool shows reasonable variability over the spectrum of the 72 nested EPAs and within the 6-point supervision scale. This variation is of importance because it is generally not seen with less aligned scales otherwise used in workplace-based assessment of postgraduate medical training [9]. This finding is now extended to undergraduate medical training and the adapted granularity of professional activities and supervision levels of the assessment tool introduced in this study.

In regard to internal consistency, the item analysis of the subscales is good for all end-of-training EPAs, except for EPA 3.1 (“Seek consent for medical procedures and diagnostics”). The role of medical students is not well defined in this area and is interfered with by legal conditions on what students are allowed to do without direct supervision. This EPA would need better clarification in further studies.

Comparisons revealed that the supervision level between the three rating groups was not significantly different for most of the EPAs. The students’ self-rating of their ability to perform a task was slightly higher than their self-assessed history in performing the task. However, the magnitude of this overrating was smaller than what we had expected, and this finding adds to the literature on students’ self-assessment [23]. With regard to the mean among all EPAs, the difference was in the range between “all findings need to be double-checked” and “only key findings need to be double-checked” after the task was self-reliantly carried out by the student. Overall, the supervisors rated the students’ ability to perform a task even slightly higher than the students rated themselves. However, we found several significant differences among the supervisors’ and students’ ratings of students’ ability to perform a task. Some were due to higher ratings by the supervisors, and others were due to higher ratings by the students.

With regard to correlations of the supervision level between the three rating groups, there appears to be a partly heterogeneous picture. Students’ self-rating of their ability to perform a task closely correlates with, and is likely grounded in their perceived experience in performing the task. The results derived by the supervisors show a good correlation in most of the EPA ratings of two supervisors for the same medical student. This correlation may indicate that the supervisors have a shared view when assessing the workplace performance of a clerkship student. It is unclear whether the shared view of supervisors is based on their own history and experiences when performing those tasks or instead reflects a general judgement approach when a particular pattern of students performance is observed [24]. The finding that students’ self-ratings do not correlate well with supervisors’ ratings at an individual level is a known phenomenon in the literature [25]. This gap can serve as a rich and tangible information source in the discussion between students and supervisors in the context of formative feedback.

Educational value represents another aspect of our utility evaluation. From the programme perspective of the extent to which educational outcomes are in fact met, we explored the percentage of students who would reach a certain level of supervision in clinical supervisors’ ratings of students’ ability to perform a task. When defining EPAs, the Charité medical faculty assumed that supervision level 5 could be achieved for all EPAs by the end of the final-year clerkship. The results of this study indicate that this level was in fact achieved by many, but not all, of the final-year students. We believe this will be a good starting point in our future attempts to improve teaching and learning at our institution so that, for instance, a benchmark of 90% or higher can be reached via these end-of-training EPAs. One must keep in mind that our institution is still in an early phase of implementing these EPAs into its curriculum [17]. The EPA assessed in this study are not yet formally adopted for the final-year clerkship and not yet explicitly communicated to clerkship students and supervisors. In turn, the results of this study may also be used to further refine our end-of-training EPAs to better represent achievable outcomes for undergraduate medical training.

Overall, the utility evaluation of our EPA-based assessment tool provides us with a sufficient basis to refine the tool and to explore it further as a formative assessment approach to steer students’ future learning. We envision that students can present their self-rating of their history and ability to perform these EPAs at the beginning of a clerkship rotation to their supervisor so that he/she can take this into account when deciding on students’ readiness for certain tasks. In addition, supervisors could use this tool to make explicit what tasks the student is allowed to perform and under which supervision level. Furthermore, students and supervisors can jointly identify gaps between students’ current abilities and the intended EPA outcomes for undergraduate medical training and, together, actively look for learning and practice opportunities to close those gaps. At the end of the rotation, ratings on this assessment tool, by students and supervisors, could document what has been archived in the rotation and serve as information sources for the educational hand-over to the next clinical placement. Using the tool in the future in a whole students cohort and in a formative manner will also provide us with the opportunity to more comprehensively evaluate the tool’s utility for assessment. This holds true especially for the tool’s feasibility, acceptability and impact on individual students’ learning.

Our study has some limitations. First, this study investigated students from a single medical school, so the findings may not be generalisable to other educational contexts. Second, the investigated sample comprised self-selected volunteering students and not randomly chosen students. This may have led to a selection bias. Third, the supervisor ratings were not fully based on direct and indirect observations of the workplace performance of medical students performing the tasks. Finally, the supervisors’ rating of students’ ability to perform a task did not implicate entrustment consequences in real life; supervisors may in fact have to be more restrictive when it comes to real patient care.

## Conclusions

In conclusion, this article introduces an assessment tool that is adjusted to the final-year clerkship context and allows us to capture the workplace performance of medical students in their final-year clerkship in relation to the standard of a full set of end-of-undergraduate-training EPAs. Its utility exploration in a group of final-year clerkship students and their supervisors suggests that its further development may serve as a formative and outcome-aligned approach to the assessment of final -year students before they enter residency.

## Additional files

Additional file 1: Supversisor version (XLSX 18 kb)

Additional file 2: Student version (XLSX 18 kb)
